# Assessment of SOX2 performance as a marker for circulating cancer stem-like cells (CCSCs) identification in advanced breast cancer patients using CytoTrack system

**DOI:** 10.1515/med-2025-1265

**Published:** 2025-08-22

**Authors:** Małgorzata Szostakowska-Rodzoś, Anna Fabisiewicz, Izabella Myśliwy, Agnieszka Jagiełło-Gruszfeld, Aleksandra Konieczna, Ewa A. Grzybowska

**Affiliations:** Department of Molecular and Translational Oncology, Maria Sklodowska-Curie National Research Institute of Oncology, Roentgena 5, 02-781, Warsaw, Poland; Oncological Clinic, Oncological Mazovian Hospital, Warsaw, Poland; Department of Breast Cancer and Reconstructive Surgery, Maria Sklodowska-Curie National Research Institute of Oncology, Warsaw, Poland

**Keywords:** circulating tumor cells, SOX2, cancer stem cells, breast cancer, CytoTrack, liquid biopsy

## Abstract

**Background:**

Recent studies have highlighted that one of the main drivers for metastatic formation and resistance to the therapy are circulating tumor cells (CTCs) and cancer stem-like cells (CSCs). Measuring the CTCs has emerged as a non-invasive procedure for selecting the patients with higher risk of progression/relapse. However, still there are no methods enabling the identification of stem-like phenotype of the CTCs.

**Methods:**

The image-based method was used for the identification of the circulating cancer stem-like cells (CCSCs) in metastatic breast cancer patients. The method was optimized using the CSCs established in the mammosphere culture of MCF-7 cells. Next the protocol was implemented to identify the CCSCs in samples collected from 60 patients.

**Results:**

The recovery ratio for CCSCs identification using the established method was ∼60%. The CCSCs were identified as rare events accruing only in 2 patients out of 60, who participated in the study. Interestingly, the CCSCs were found only in CTC clusters. The analysis of SOX2 expression in formalin-fixed, paraffin-embedded material, revealed that the SOX2 expression was present in primary tumor samples.

**Conclusion:**

The CCSCs presence was found to be a very rare event. The obtained results suggest that the CCSCs are mainly present in CTC clusters and stem-like reprograming of the cancer cells might occur early, in the primary tumor.

## 1 Introduction

Breast cancer (BC) is one of the most common female tumors diagnosed worldwide. In the US, BC represents 29% of all new cancer cases in women [1]. Biological subtypes of BC are classified according to the expression of steroid receptors (estrogen [ER) and progesterone [PR]) and human epidermal growth factor receptor 2 (HER2). Cancers that are positive for ER receptor (ER+) and/or PR receptor (PR+) are classified as luminal [2]. Luminal BC (ERα+) is known to have a more favorable prognosis in terms of 10-year survival than other subtypes. However, 10-year survival does not fully reflect the long-term survival and mortality of these cancers. Therefore, identifying the most effective diagnostic markers for the long-term prognosis is crucial in these malignancies.

Current predictive oncology research works are focused on non-invasive biomarkers for cancer recurrence and survival perdition. Most studies concentrate on analyzing blood markers like inflammation-based markers, albumin-to-globulin ratio or carbohydrate antigens [3,4] that had shown independent prediction value for various cancers, like colorectal cancer. However, as BCs, especially luminal BCs are considered “cold” inflammation malignancies, the utility of inflammation based-markers remains limited for specific subtypes of BC [5]. Other classical markers like cancer carcinoembryonic antigen and CA15-3, shown to be often associated with disease recurrence and metastasis [6] in BC, they have their limitations [7]. Another approach, represented by modified Glasgow prognostic score designed to assess the systemic inflammatory response, may further improve prognostic efficiency and has been shown to have an independent prognostic value in BC patients [8]. Comparing such classic serum-based indices with novel biomarkers like circulating tumor cells (CTCs)/circulating cancer stem-like cells (CCSCs) may further refine risk stratification strategies and guide personalized treatment approaches.

Precise prognosis for the patients with luminal BCs is difficult, because late relapses are common. Late relapse in a woman with ERα+ BCs is associated with the activation of dormant tumor cells at metastatic sites [9]. Dormant tumor cells display growth arrest that precludes proliferation, which lowers their sensitivity to the cytotoxic treatment. The presence of dormant, disseminated tumor cells (DTCs) has been shown to be associated with poorer prognosis, with 40–60% of patients with DTCs detected in bone marrow suffering from metastatic disease [10,11]. Thus, the detection of DTCs may be important for prognosis. However, the main challenge is the ability to correctly assess probability of DTCs reactivation, leading to metastatic progression.

One of the potential triggers of this process might be the cells’ reprogramming associated with the generation of cancer stem-like cells (CSCs). CSCs are a small population of tumor cells that are capable of self-renewal, producing heterogeneous lineages of cancer cells that comprise the tumor. CSCs are a unique biological subpopulation of cancer cells that could survive indefinitely [12]. One of the hallmarks of CSCs is the expression of stemness markers: SOX2, OCT4, and NANOG. From these markers, SOX2 is one of the most well-known for its association with the progression of cancers. It is known that SOX2 is a regulator of the pathways that regulate epithelial to mesenchymal transition, such as NF-κB, WNT, NOTCH, HEDGEHOG [13,14,15]. Despite growing knowledge about CSCs’ importance in cancer, it is still unclear at what stage of cancer progression these cells arise. However, it is suspected that because of the ability for self-renewal and high epithelial-mesenchymal plasticity, the stem-like phenotype might be present more in CTCs. Most of CTCs occur as circulating single cells; however, they might also appear as multicellular (>3 cells) groups, called clusters. CTC clusters have been shown to be even ten times rarer events than single CTCs, but most probably they have higher metastatic potential [16]. The detailed mechanism of CTC clusters origin and their cellular component is still unknown. Some studies have shown that CTC clusters might be somewhat a reservoir for CSCs as CD44 expression [17] and hypomethylation of stemness markers [18] have been observed in CTC clusters.

As the cancer-stem like cells are also known from their properties to form new metastases and drug resistance, it is believed that some fraction of CTCs might harbor the stemness properties and be classified as the CCSCs. However, the details of CCSCs’ metastasis potential and clinical value are still unknown. One of the most challenging issues in CCSCs characterization and studies is lack of validated methods for CCSCs identification. Despite the growing knowledge and the advancement in CTCs research, the methods for CSCs identification are still missing. Currently only few methods describing CCSCs were described: nanoparticle-mediated Raman imaging method for CCSC characterization [19], separation enrichment [20], and flow cytometry [21].

Here we report the new results on CCSCs identification in advanced BC patients, based on the SOX2 expression. We modified previously described protocol for CTCs detection [22] for identification of the SOX2-positive CTCs, as representatives of CCSCs. In this work, we present optimization of this method with the use of MCF-7-derived CSCs using CytoTrack system, as well as the pilot studies on patients’ samples.

## 2 Materials and methods

### 2.1 Cell culture

The MCF-7 cell line was chosen for this model as it is well-established luminal BC model with known tendency to from mammospheres with SOX2+ cells. MCF-7 cells (ATCC, passage 20–30) were cultured in DMEM Complete medium (Merck) with 10% heat-inactivated FBS, 100 µg/mL penicillin, and 100 µg/mL streptomycin. For spheroid culture of MCF-7 cell line, the single cell suspensions were seeded in density 4 × 10^4^ cells/well into 6-well Corning Costar Ultra-Low attachment plates in 3dGRO Spheroid Medium (Merck) with 100 µg/mL penicillin and 100 µg/mL streptomycin. The single cell suspensions were made by filtering the cell culture with cell strainer of 40 µm. Spheroid culture was set in a starting volume of 2 mL of the medium. The half culture volume of fresh medium was added every 3 days. Sphere formation and stemness markers were evaluated after 7 days of sphere culture.

### 2.2 Reverse transcription qPCR (RT-qPCR)

The expression of the stemness marker *SOX2* in mammosphere culture was confirmed by the RT-qPCR. After 7 days of sphere culture cells from mammosphere, 2D cell culture was harvested in the same time-points at the confluency of ∼70%. The RNA was isolated with the use of Universal RNA Purification Kit (EURx) according to the manufacturer’s protocol. 1 µg RNA was reverse transcribed with the use of NG dART RT kit (EURx) according to the manufacturer’s protocol. The success of the RT-PCR reaction was confirmed with the PCR reaction for *GAPDH* with the use of starters: forward 5′-GAAGGTGAAGGTCGGAGTC-3′; reverse 5′-GAAGATGGTGATGGGATTTC-3′. The reaction conditions were set as: 95^o^C for 5 min, followed by 25× cycles: 95°C for 30 s, 60°C for 30 s, 72°C for 30 s, ending with 72°C for 5 min and 4°C incubation. The qPCR reaction was performed in Applied Biosystems™ 7500 Fast (Applied Biosystems) thermocycler. The PCR reaction was set in 20 µL containing 100 ng cDNA, 2× TaqMan™ Gene Expression Master Mix (Applied Biosystems), ddH_2_O, 20× TaqMan Gene Expression assays: *SOX2* (ThermoFisher, Hs04234836_s1) and *GAPDH* (ThermoFisher, Hs02786624_g1). The reaction conditions were: 95^o^C for 10 min followed by 45× cycles of 95°C for 30 s, 60°C for 60 s. All data with Ct value lower than 25 for *GAPDH* reference gene were excluded from the analysis. All samples were run in triplicates. Wells with high SD (>0.3) were excluded from the analysis. The change in *SOX2* expression was calculated using ΔΔCt.

### 2.3 Biological sample collection

Sixty patients diagnosed with luminal metastatic BC (MBC) were selected for the study. The inclusion criteria for patients were: age >18, ongoing hormonal treatment, and identification of distant metastases. All participants signed the informed consent.

For spike-in experiments blood was collected from healthy volunteers. Formalin-fixed, paraffin-embedded (FFPE) primary cancer samples were collected from the archive of the Pathology Department in National Research Institute of Oncology. The study protocol was approved by the National Research Institute of Oncology Ethics Committee (84/2020).

### 2.4 Spike-in experiments

For optimization of the detection of the SOX2 positive cells, we calibrated the sample processing protocol using MCF-7 cells from 2D and mammosphere culture. The cells were counted and 100 cells were suspended in blood sample from healthy donor. The recovery ratio for identification of spiked cells was calculated as: \[\frac{{\mathrm{identified\; cells}}}{{\mathrm{spiked}}-{\mathrm{in\; cells}}}\times 100 \% ]\] . The spike-in experiments were done in duplicates. The previously optimized protocol for CTCs identification [22] was used as the basis for the further modification and optimization of the final conditions. The conditions pre- and post- optimization are listed in Table S2.

### 2.5 Sample processing

Blood samples were collected in the 9 mL EDTA tubes and processed within 2 h after collection. Samples were centrifuged at 2,500 × *g* for 10 min. The buffy coat containing nuclear cells, including tumor cells, were transferred to a new 15 mL tube. The residual erythrocytes were lysed with the use of FACS Lysing solution (BD Biosciences) and with incubation time 15 min in the room temperature. Then, the samples were centrifuged at 3,000 × *g* for 5 min. The lysis step was repeated, with the incubation time 10 min in room temperature and centrifugation at 3,000 × *g* for 5 min. Thereafter, cells were permeabilized with the saponin-based permeabilization buffer (0.5% saponin + 0.5% BSA + 0.01% sodium azide in 1× PBS), with incubation time 15 min in the room temperature followed by centrifugation at 3,000 × *g* for 5 min. Permeabilized cells were stained with the use of Alexa Fluor 488-conjugated pancytokeratin (pan-CK) antibody (final dilution 1:25), AlexFluor700 conjugated CD45 antibody (final dilution 1:50), PE conjugated E-Cadherin antibody (final dilution 1:50), eFluor660-conjugated SOX2 (final dilution 1:12), and 4,6-diamidino-2-phenylindole (DAPI) (final dilution 1:1,000). The antibodies stain was diluted using the perm/wash buffer (0.25% saponin + 1% BSA in 1× PBS). Samples were left in the antibodies over-night in the dark, at 4°C. Next stained cells were washed two times with perm/wash buffer, containing 1% BSA and centrifugated at 3,000 × *g* for 5 min. Washed and stained cells were resuspended in 1 mL of ultra-pure H_2_O and smeared on the glass disc in sterile conditions. The discs were left to dry overnight. Dried samples were mounted with glycerol-based mount medium (80% glycerol in Tris-HCl, pH = 8.5 with 0.5% N-propyl gallate) and fixed with the use of fixogum. Prepared samples were stored in −80°C until CytoTrack analysis. Detailed materials, including buffer solutions and antibodies source are listed in Table S1.

### 2.6 CTCs identification

All samples were processed as described above and analyzed on CytoTrack system. For analysis the glass disc containing stained cells was mounted in the mounting arm with a spring-lock mechanism. Focus plan was obtained based on the DAPI channel, at eight places on the disc. Scanning was performed with 488 nm Argon-Neon laser, in a spiral pattern with a bandwidth of 10μm, a process taking ∼5 min. All signals from the Alexa Fluor 488 emission channel were recorded and listed in the hotspot table. Recorded events were visually inspected by the operator. The criteria for CTC identification were set as: nearly round size with ≥6 µm diameter, visible nucleus, pan-CK signal, and negative surface stain for CD45. Detected cells with the above criteria were identified as CTCs. Clusters were defined as group of ≥3 cells, with at least three visible nuclei in DAPI channel, with at least three cells identified as CTCs. For SOX2+ CTCs identification, we established the following criteria: nearly round size with ≥6 µm diameter, visible nucleus, pan-CK signal, positive surface stain for E-cadherin, and positive nuclei for SOX2. Cells matching these criteria were defined as SOX2+ CTCs.

### 2.7 Immunohistochemistry (IHC)

IHC staining was performed on 3-μm FFPE tissue sections using Envision Detection System (DAKO, Glostrup, Denmark). Tissue sections were deparaffinized with xylene and rehydrated in a series of ethanol solutions of decreasing concentration. Heat-induced epitope retrieval was applied by incubating the samples in Target Retrieval Solution pH 6 (DAKO) in a 96°C water bath, for 30 min. Cooled slides were treated with a Blocker of Endogenous Peroxidase (DAKO) for 5 min and then incubated with the primary rabbit monoclonal antibody SOX2 (C70B1, Cell Signaling Technology) in dilution 1:100 for 1 h in room temperature. The color reaction was developed with 3,3′-diaminobenzidine tetrahydrochloride (Dako) as a substrate. Nuclear contrast was achieved by using hematoxylin counterstaining.

### 2.8 Statistical analysis

The categorical data generated in the study were analyzed via U-Mann Whitney or Kruskal–Wallis non-parametric tests. The primary end point was overall survival (OS) in relation to CTCs presence. The OS was defined as the time from the date of blood collection to death from any cause. If an outcome was not reached during the observation time, the variables were censored. Kaplan–Meier plots and the log-rank tests were used to illustrate and compare survival between subgroups.


**Ethics statement:** The study was conducted in accordance with the Declaration of Helsinki, and approved by the Ethics Committee of the Maria Sklodowska-Curie National Research Institute of Oncology (84/2020). All participants read and signed the informed consent.

## 3 Results

### 3.1 Identification of the SOX2+ tumor cells

**Figure 1:**
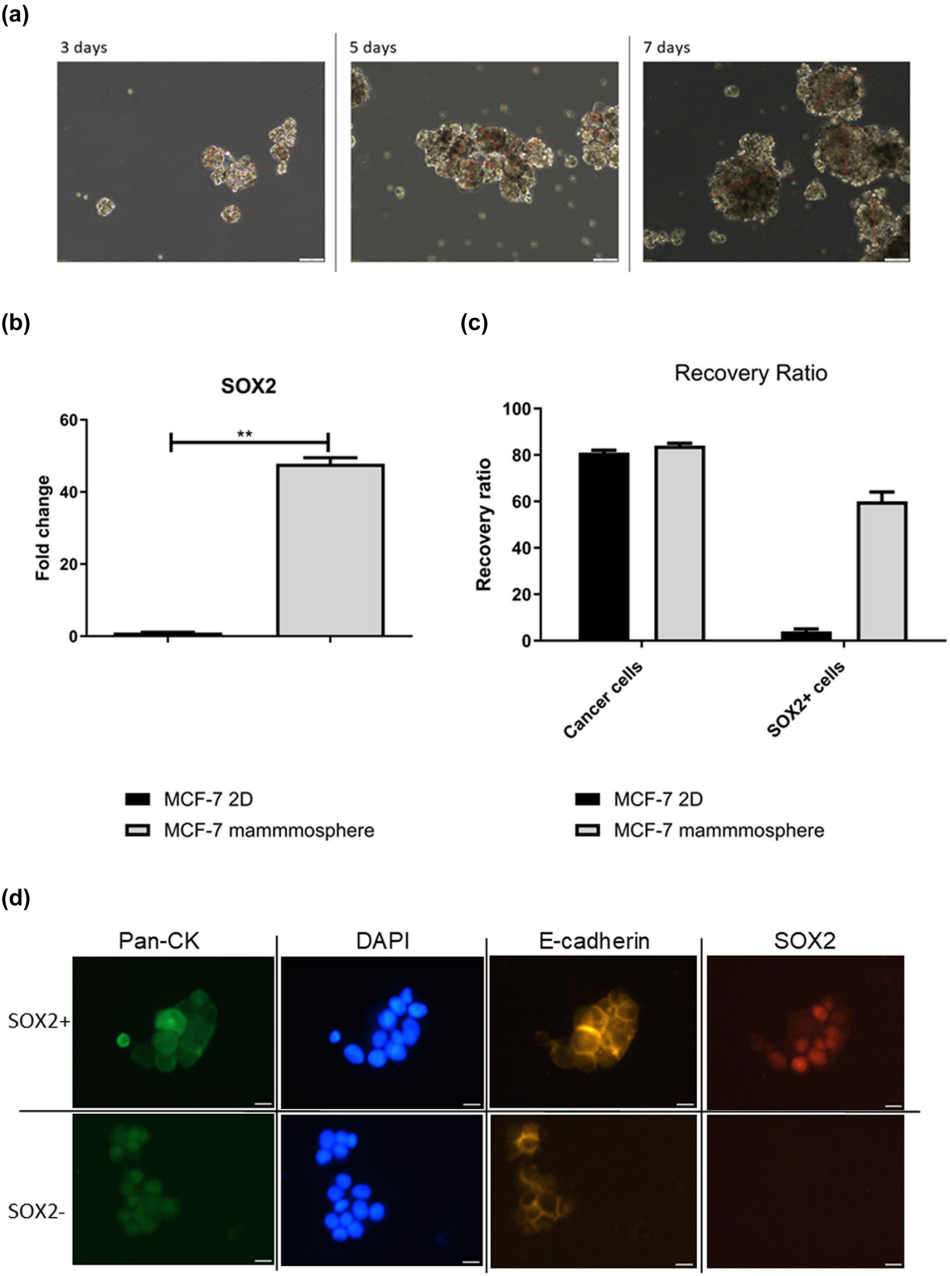
The results from sphere formation assay and recovery ratio for identification of SOX2+ cells in blood sample. (a) The microscopic images of MCF-7-derived spheres in different time-points of the mammosphere formation assay. (b) The expression of *SOX2* measured via qPCR on 7th day of sphere formation; **p-value <0.01. (c) The recovery ratio of panCK + cells (tumor cells) and SOX2+ tumor cells determined during the spike-in experiments. (d) The images from CytoTrack system with identified SOX2+ cells in spike-in experiments.

The spheres were evaluated every 2 days to check for the overgrowth of the sphere (>200 µm diameter). The spheroids were passaged every 14 days. The mean sphere diameter on the 7th day of culture was ∼173 µm (Figure 1a). The *SOX2* expression was measured on the 7th day of the spheroid culture, as previously we identified this time-point as the most optimal for *SOX2* evaluation [23]. The elevated *SOX2* expression in cells was confirmed via qPCR (Figure 1b). Next, the cells with confirmed SOX2 expression were used for the CCSCs identification optimization.

The cells from cell cultures: SOX2-culture (2D) and SOX2 + culture (mammosphere) were used in the spike-in experiments and optimization of the protocol. During the optimization process the most challenging was identification of the SOX2 staining as antibodies tend to not penetrate the nucleus. Therefore, the proper parameters of the permeabilization buffer and permeabilization time were optimized. Further optimizations included antibodies dilution, incubation time, and conditions. The final protocol described in the methods section was chosen as the one with the best performance. Using this protocol the recovery ratio for spike in experiments was as follows:

81% for MCF-7 2D cells and 84% for MCF-7 mammosphere cells. The mean recovery ratio for SOX2+ cells identification was: 4% for MCF-7 2D cells and 60% for MCF-7 mammosphere cells (Figure 1c and d). The low recovery ratio of the SOX2+ cells might be associated with the necessity of determining the stain location (nuclear).

### 3.2 Patients characteristics

Overall, 60 patients were enrolled in the study; however, samples from 5 patients were discarded due to the technical issues. Therefore, in the final analysis, data from 55 patients were used. The median age of patients was 66 (range 35–84), all patients were previously treated with antiestrogen therapy, 69% of patients received radiotherapy and >80% of patients were treated with the CDK4/6 inhibitors and chemotherapy as adjuvant therapy. The detailed patients group characterization is presented in Table 1.

**Table 1:** Clinical characteristics of the patients group

**Age**	
<66	27
≥66	28
**HER2 status**	
HER2+	4
HER−	49
N/D	2
**No. of meta sites**	
1	30
2	16
≥3	9
**Meta sites**	
Bones	37
Liver	15
Lung	14
Other	17
**Histological subtype**	
NST	41
Lobular	5
Other	9
**Treatment**	
HTH	4
HTH + CHTH	7
HTH + CDK4/6	7
HTH + CHTH + CDK4/6	37
**Radiotherapy**	
RTH+	38
RTH−	17

### 3.3 CTC identification and SOX2 detection in patient samples

**Figure 2:**
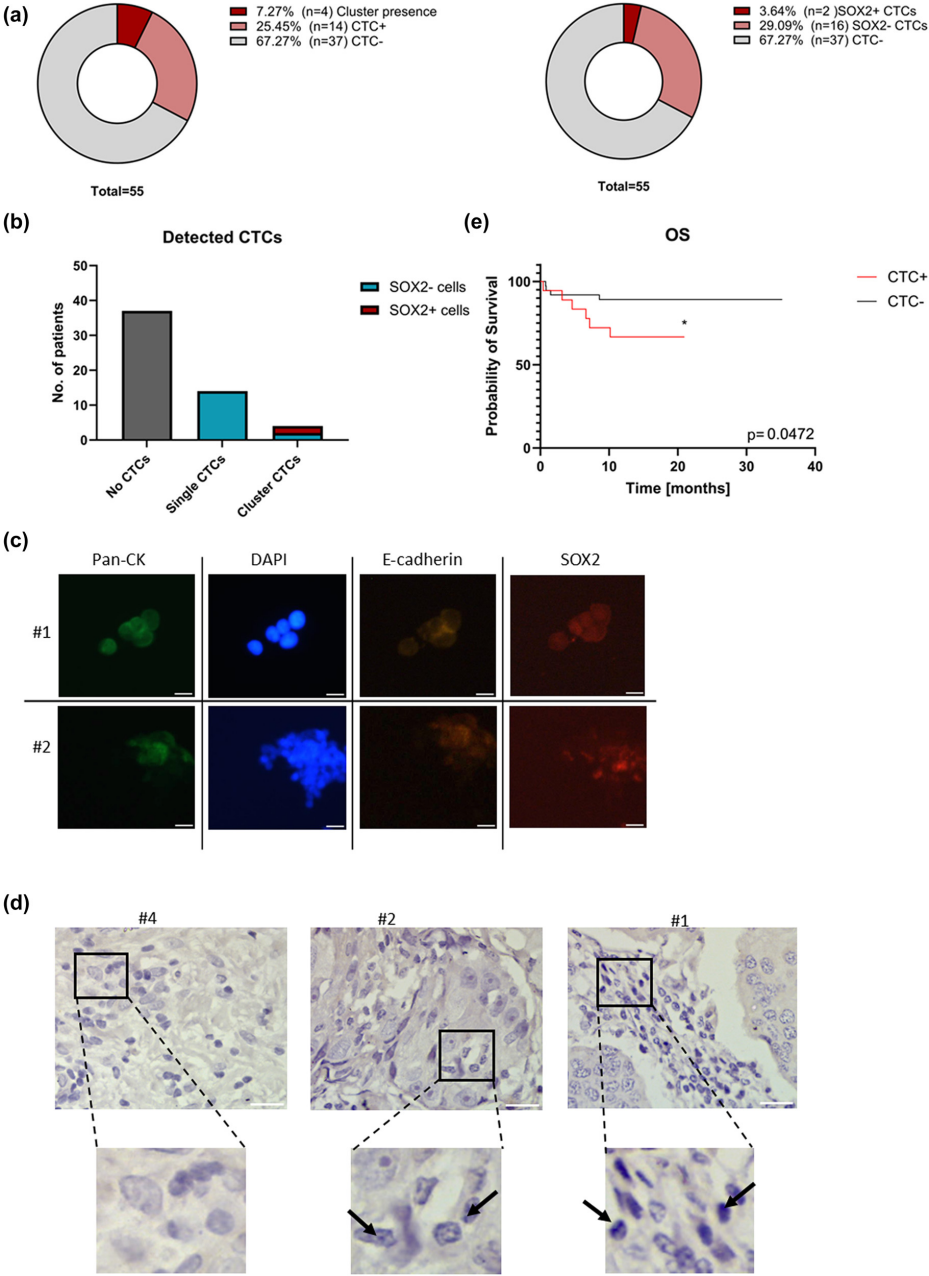
The consolidated data from CTCs detection, where (a) The frequency of CTCs+ patients with detailed CTC clusters positive patients (left panel) and detailed SOX2+ CTCs patients (right panel). (b) The number of patients with SOX2+ CTCs detected; (c) The SOX2+ detected cells in patients; the patient number is stated on the left: #1 and #2. (d) The SOX2 expression detected in the FFPE samples of patients: patient #4 SOX2 negative sample; patient #2 SOX2 positive with low SOX2 expression (red arrows); patient #3 SOX2 positive patient (red arrows). (e) The prognostic value of the detected CTCs for the OS.

The CTCs were detected in ∼33% of advanced BC patients. The CCSCs, defined as CTCs with detectable SOX2 expression, were detected in samples from two patients (Figure 2b). These CCSCs were only detected in CTC clusters, suggesting the importance of CTC clusters in CSCs generation. The CTC clusters were detected in four patients.

The primary tumor samples FFPEs were collected for 40 patients included in the studies. For 15 samples without CTCs in blood sample, the FFPE samples were not available. The SOX2 protein expression was found to be a very rare event. Overall, we detected very low nucleus expression of SOX2 in samples derived from two patients. Interestingly, these patients were also identified with the SOX2+ CTCs and clusters. This might suggest that CSCs transformation occurs early in the cancer progression and this phenotype might be maintained during progression.

As the potential CCSCs were detected only in two patients, the data are not suitable for survival analysis considering the CCSCs value in the BC prognosis. The CTCs presence was identified as a significant prognostic marker for OS (Figure 2e), which complies with the majority of research.

## 4 Discussion

Since the current tools for long-term prognosis in luminal BC patients lack the required precision, there is a need for more universal approach and the identification of the new non-invasive biomarkers.

In this report, we presented a novel method for CCSCs identification based on previously described CytoTrack system [22,24]. This EpCAM independent system for CTCs identification proved to be a useful tool in liquid biopsy research [22]. It has two advantages over the other systems based on immune detection: it does not eliminate any cells and it allows for the additional recognition of the markers other than standard CTC detection. In the approach presented here, this possibility was utilized to recognize stem cell marker SOX2, which represents an alternative for the surface markers used so far (CD133, CD24, and CD44) [25,26,27].

The recovery ratio for the SOX2+ cells detection in this study was low (∼60%). This might be associated with the specific mechanisms behind CSCs generation in mammosphere culture. Despite the overall upregulation of the *SOX2* expression, it is possible that in the mammosphere model, only a fraction of cells transforms into CSCs. The other reason for relatively low recovery ratio of CCSCs via this method might be the technical difficulty associated with nuclear staining for SOX2. Thus, further optimization of the staining presents the opportunity for detecting larger population of SOX2+ cells.

In our study, we observed CCSCs identified as SOX2+ as the rare events occurring only in two patients (3.64%). In contrary, some previous studies describe CCSCs as more frequent for advanced BC patients, e.g., in ∼44% [25] of MBC patients. However, the other study was using different stem marker (CD133) for CCSCs identification. It was described before that while SOX2 and CD133 are both considered stemness markers, their expression is independent, and their clinical relevance might differ. SOX2 expression was found to be positively correlated with the regional lymph nodes metastasis, while the same was not found for CD133 expression [28]. Interestingly, while analyzing samples from patients, we detected SOX2 expression only in CTC clusters. Our data reporting SOX2 expression exclusively in CTC clusters confirm some previous findings concerning CTC clusters, reported by Gkountela et al. [18], who analyzed the methylation profiles of single and cluster CTCs. They observed that binding sites for stemness- and proliferation-associated transcription factors are specifically hypomethylated in CTC clusters, including binding sites for SOX2 [18]. This change in methylation should result in activation and expression of SOX2 specifically in CTC clusters. Other studies also reported that population of CSCs is found in CTC clusters [17]. These studies, as well as our data might suggest that the CTC clusters are the main reservoir of CSCs in cancer progression. It is also worth to point out that when cells are grown in non-adherent conditions, forming mammospheres, which bear some resemblance to CTC clusters, they display elevated expression of stem cell markers. These properties may be important for the survival of tumor cells in the harsh conditions of the bloodstream. Further studies addressing this issue with large group of patients should be done for better understanding of clusters’ role in CSCs maintenance.

Interestingly, IHC studies of the matching primary tumor samples revealed SOX2 presence only in the samples from patients who also had SOX2 + CTC clusters, suggesting that CSCs marker expression occurred early in cancer progression.

Overall, our studies highlight the difficulty of identification of the CCSCs in blood samples from advanced BC patients using SOX2 as a marker. Nevertheless, we determined that probable reservoir for CSCs are CTC clusters, highlighting the importance of further studies associated with the clinical value of CTC clusters on large cohort of patients.

We identified small group of patients and low recovery ratio for CCSCs as the main limitations of our studies.

In conclusion, novel biomarkers like CTCs/CCSCs offer a promise for better prognosis, but need further development. Additionally, their combined use with other markers, like inflammation-based score determination may further improve prognosis [29].

## Supplementary Material

Supplementary Table
