# Supplementary material for: Assessment of SOX2 performance as a marker for circulating cancer stem-like cells (CCSCs) identification in advanced breast cancer patients using CytoTrack system
Source: Open Med (Wars). 2025 Aug 22;20(1):20251265. doi: 10.1515/med-2025-1265 (PMC12413790; doi:10.1515/med-2025-1265)
Supplement: Supplementary Table [file med-2025-1265-sm.pdf]

Supplementary material

Table S1: The materials used for the final protocol, for CCSCs identification

Material	Cat. No.	Producer
FACS lysis solution	349202	BD Biosciences
Saponin	S4521-10G	Merck
Bovine Serum Albumin	A9418-50G	Merck
Glycerol	1040950250	Merck
N-propyl gallate	P3130-100G	Merck
Pan Cytokeratin (AE1/AE3), Alexa Fluor™ 488	53-9003-82	ThermoFisher
CD45 (HI30), Alexa Fluor™ 700	MHCD4529	ThermoFisher
E-cadherin (67A4), PE	A15784	ThermoFisher
SOX2 (Btjce), Alexa Fluor™ Plus 647	740013TP647	ThermoFisher
DAPI (4′,6-Diamidine-2′-phenylindole dihydrochloride)	10236276001	Merck
**Buffer**	**Components**	
Perm/Wash buffer	0.5% saponin + 0.5% BSA + 0.01% sodium azide in 1xPBS	
Mount Medium	80% glycerol in Tris-HCl pH=8.5 with 0.5% N-propyl gallate	

Table S2: The conditions for the staining pre- and post- optimization. Main changes were associated with the staining time and SOX2 antibodies concentration. The staining time was prolonged for higher reproducibility and recovery ratio for nuclear staining of SOX2. Antibody concentration was reduced compared to the first protocol, because of the high background

Staining conditions pre-optimization	
Permeabilization time	10 min
Staining time	60 min, 4°C
panCK dilution	1:25
CD45 dilution	1:50
E-cadherin dilution	1:50
SOX2 dilution	1:6
DAPI dilution	1:1,000
**Staining protocol post-optimization**	
Permeabilization time	15 min
Staining time	O/N, 4°C
panCK dilution	1:25
CD45 dilution	1:50
E-cadherin dilution	1:50
SOX2 dilution	1:12
DAPI dilution	1:1,000
